# Supplementary material for: Effects of Maternal Pre-Pregnancy BMI on Preterm Infant Microbiome and Fecal Fermentation Profile—A Preliminary Cohort Study
Source: Nutrients. 2025 Mar 11;17(6):987. doi: 10.3390/nu17060987 (PMC11944810; doi:10.3390/nu17060987)
Supplement: Supplementary file 1 [file nutrients-17-00987-s001.zip › nutrients-3497197-supplementary.pdf]

SUPPLEMENTARY FIGURES

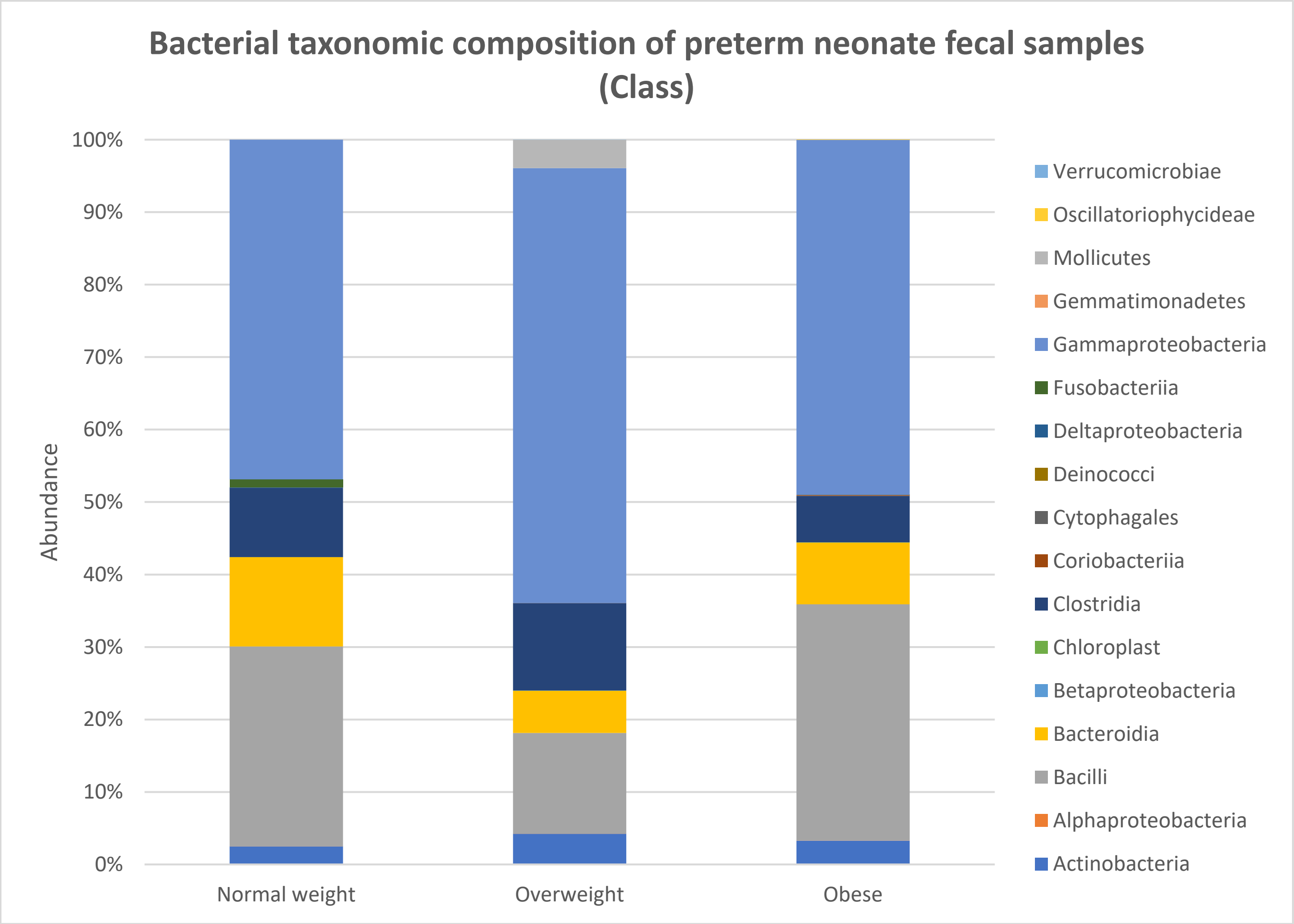

S1a

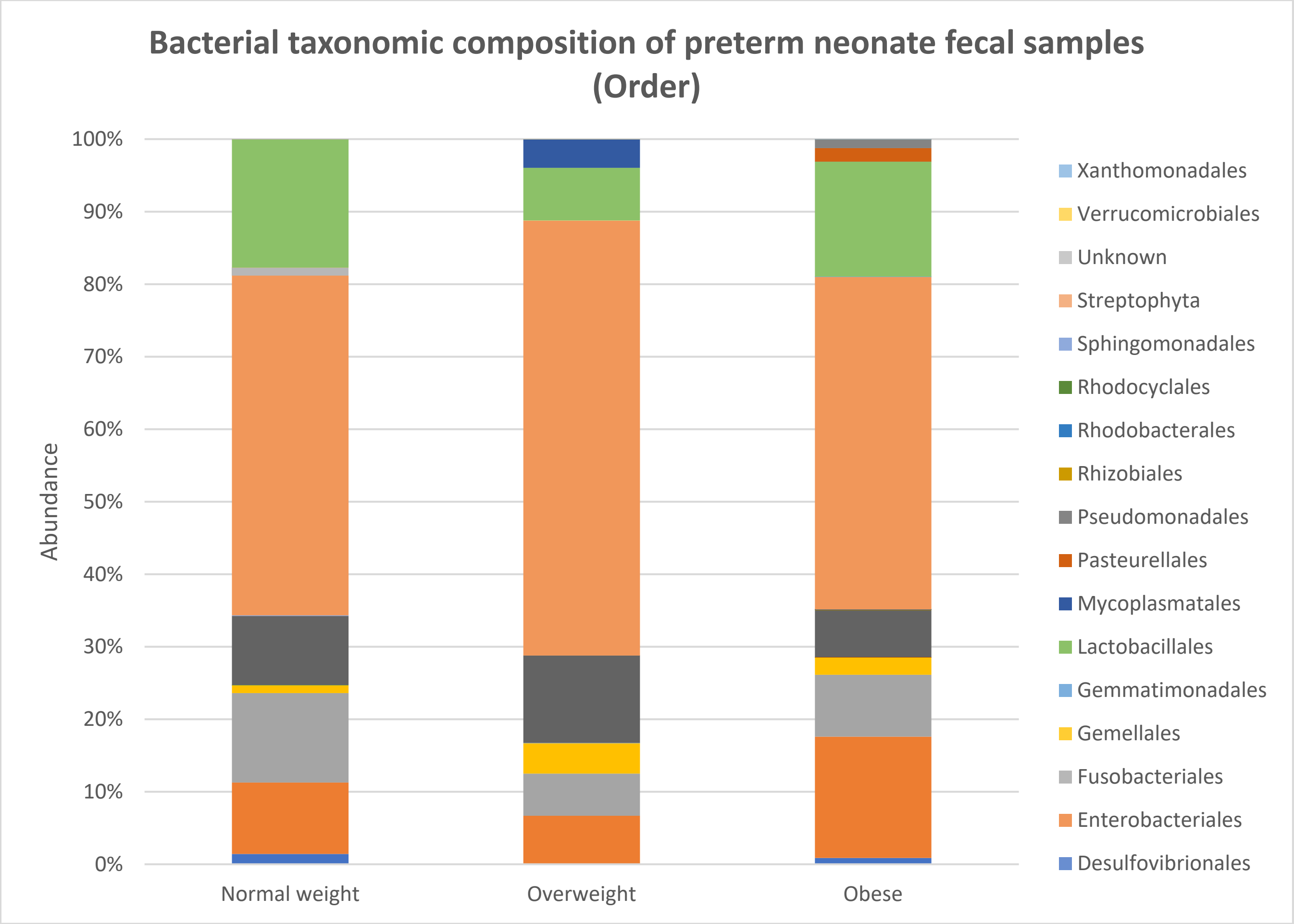
Bacterial taxonomic composition of preterm neonate fecal samples (Order)
100%
90%
80%
70%
60%
50%
40%
30%
20%
10%
0%
Abundance
Normal weight
Overweight
Obese
Xanthomonadales
Verrucomicrobiales
Unknown
Streptophyta
Sphingomonadales
Rhodocyclales
Rhodobacterales
Rhizobiales
Pseudomonadales
Pasteurellales
Mycoplasmatales
Lactobacillales
Gemmatimonadales
Gemellales
Fusobacteriales
Enterobacteriales
Desulfovibrionales

S1b.

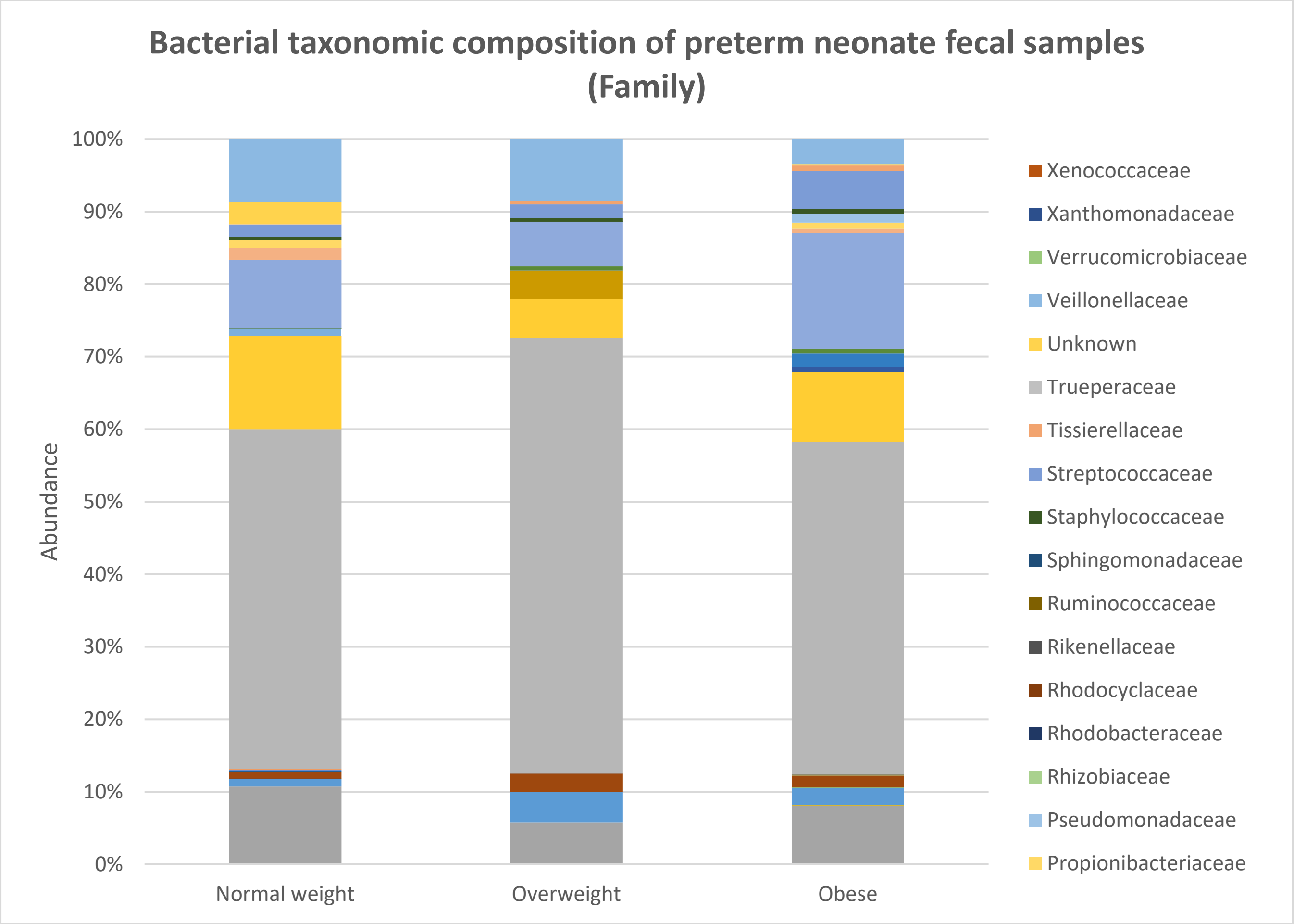

Bacterial taxonomic composition of preterm neonate fecal samples (Family)
100%
90%
80%
70%
60%
50%
40%
30%
20%
10%
0%
Abundance
Normal weight
Overweight
Obese
Xenococcaceae
Xanthomonadaceae
Verrucomicrobiaceae
Veillonellaceae
Unknown
Trueperaceae
Tissierellaceae
Streptococcaceae
Staphylococcaceae
Sphingomonadaceae
Ruminococcaceae
Rikenellaceae
Rhodocyclaceae
Rhodobacteraceae
Rhizobiaceae
Pseudomonadaceae
Propionibacteriaceae

S1c.

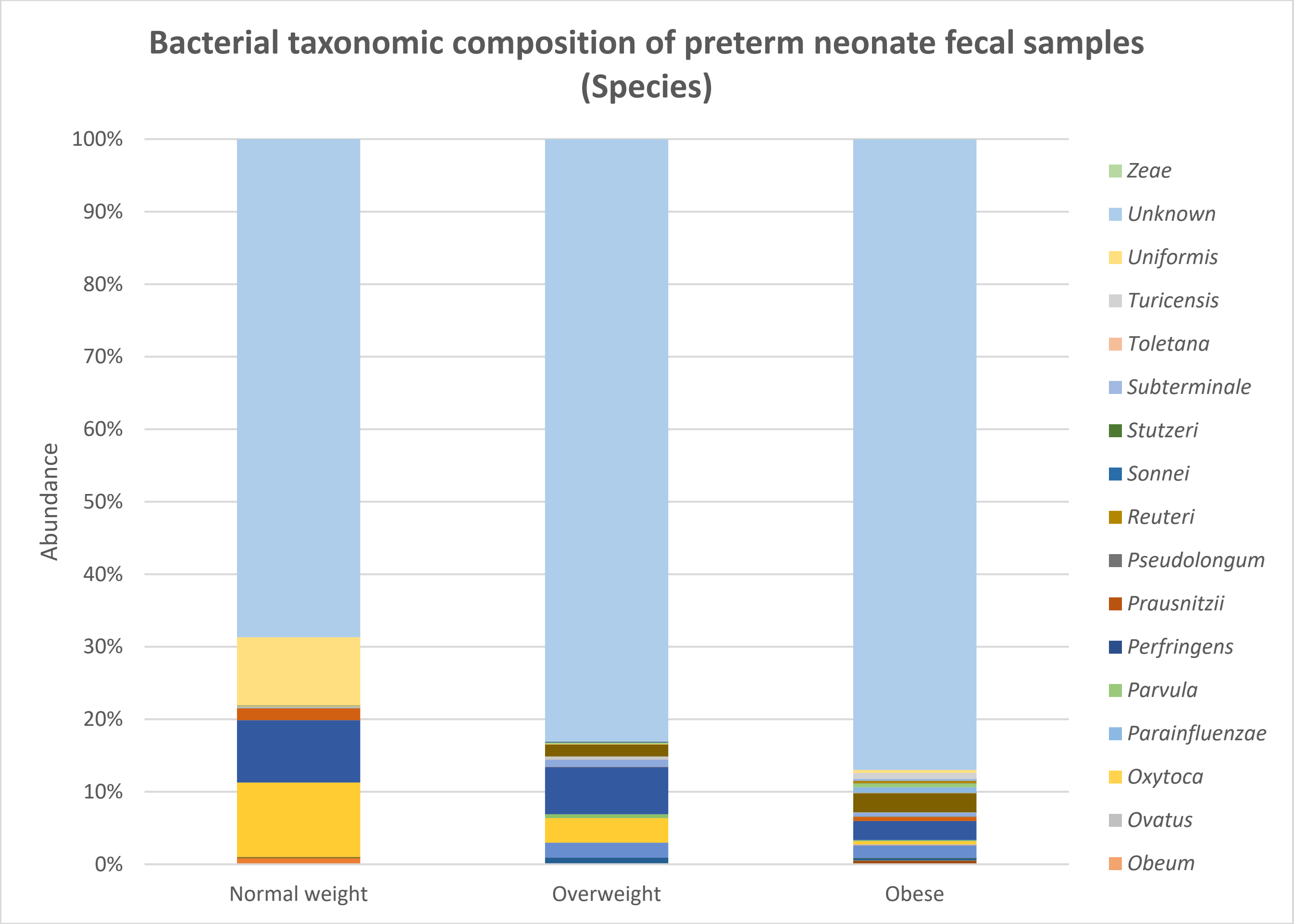

S1d.

Supplementary Figure S1. Relative abundance of microbiomes of premature neonates grouped by maternal pre-pregnancy BMI microbial population at the (a) class, (b) order, (c) family, and (d) species level.
